# Minimally Invasive Direct Thoracic Interbody Fusion (MIS-DTIF): Technical Notes of a Single Surgeon Study

**DOI:** 10.7759/cureus.699

**Published:** 2016-07-18

**Authors:** Hamid Abbasi, Ali Abbasi

**Affiliations:** 1 Tristate Brain and Spine Institute; 2 Trinity College, University of Cambridge

**Keywords:** operative surgical procedures, minimally invasive spine surgery, spinal fusion, thoracic spine, disk disease, interbody fusion, spine surgery

## Abstract

**Background:**

Minimally invasive direct thoracic interbody fusion (MIS-DTIF) is a new single surgeon procedure for fusion of the thoracic vertebrae below the scapula (T6/7) to the thoracolumbar junction. In this proof of concept study, we describe the surgical technique for MIS-DTIF and report our experience and the perioperative outcomes of the first four patients who underwent this procedure.

**Study design/setting:**

In this study we attempt to establish the safety and efficacy of MIS-DTIF. We have performed MIS-DTIF on six spinal levels in four patients with degenerative disk disease or disk herniation. We recorded surgery time, blood loss, fluoroscopy time, complications, and patient-reported pain.

**Methods:**

Throughout the MIS-DTIF procedure, the surgeon is aided by biplanar fluoroscopic imaging and electrophysiological monitoring. The surgeon approaches the spine with a series of gentle tissue dilations and inserts a working tube that establishes a direct connection from the outside of the skin to the disk space. Through this working tube, the surgeon performs a discectomy and inserts an interbody graft or cage. The procedure is completed with minimally invasive (MI) posterior pedicle screw fixation.

**Results:**

For the single level patients the mean blood loss was 90 ml, surgery time 43 minutes, fluoroscopy time 293 seconds, and hospital stay two days. For the two-level surgeries, the mean blood loss was 27 ml, surgery time 61 minutes, fluoroscopy time 321 seconds, and hospital stay three days. We did not encounter any clinically significant complications. Thirty days post-surgery, the patients reported a statistically significant reduction of 5.3 points on a 10-point sliding pain scale.

**Conclusions:**

MIS-DTIF with pedicle screw fixation is a safe and clinically effective procedure for fusions of the thoracic spine. The procedure is technically straightforward and overcomes many of the limitations of the current minimally invasive (MI) approaches to the thoracic spine. MIS-DTIF has the potential to improve patient outcomes and reduce costs relative to the current standard of care. We are currently expanding this study to a larger cohort and recording long term outcomes and costs.

## Introduction

Lower back pain is one of the most prevalent and expensive health conditions in the Western world with up to 80% of the population suffering from it at some point during their life [1]. It is the leading cause of activity limitation for people under the age of 45 and one of the most common causes of health care utilization in the United States. Spinal fusions are a common treatment for back pain caused by degenerative disk disease and disk herniation and is now the third most common surgical procedure in the United States [1]. For this reason, most of the MI spinal fusions are developed and increasingly being performed mostly for lumbar spine [2-8]. These procedures are not transferable to thoracic spine even though they reduce surgery time, blood loss, length of hospital stay, infection rates [9-10] and cost [2] compared to open procedures.

Lumbar MIS techniques cannot be applied to the thoracic spine because of anatomical considerations like the size of the pedicles, angle of the pedicles to the vertebral body, facet direction, and obstruction by the ribcage and thoracic cavity [11]. The current commonly performed thoracic procedures are open surgeries with a posterior approach or lateral approach with thoracotomy. However, the posterior approach limits any traction on the thoracic cord and requires extensive bone resection with facetectomy, as well as removal of the head of the rib in order to reach the disk space [9, 12-14].

Some MI posterior approaches to the thoracic spine have been performed under visual endoscopic guidance [15], but these surgeries have significant technical limitations and are difficult to master [16]. Here, we report our experience with a novel surgical approach that places an interbody graft or a cage through the interpleural space without direct visualization, using biplanar fluoroscopic imaging and electrophysiological monitoring.

This new single surgeon procedure is called minimally invasive direct thoracic interbody fusion (MIS-DTIF). The instrumentation in MIS-DTIF follows the same principal technique as during oblique lateral lumbar interbody fusion (OLLIF) that we described previously [17]. As in the case of OLLIF, MIS-DTIF is complemented with MI posterior pedicle screw fixation. In this study, we describe the surgical technique for MIS-DTIF and present perioperative outcomes of the first four MIS-DTIF cases for proof of concept (POC).

## Materials and methods

### Study design

This study included four MIS-DTIF patient cases for POC and to establish feasibility. The study was performed under the oversight of the Pearl Institutional Review Board (Indianapolis, IN 46260; 16-TRIS-103). Extensive consent was obtained from all patients. The surgeries were performed at Douglas County Hospital, Riverview Hospital, and Fairview Ridges Hospital.

### Patient selection

Preoperative imaging included a magnetic resonance imaging (MRI) scan, an x-ray of the thoracic spine with flexion and extension, a discogram, and a computerized axial tomography (CAT) scan. MIS-DTIF is indicated for thoracic disc herniation and degenerative disk disease after conservative therapy has failed. All patients underwent a full course of conservative therapy before being considered as a surgical candidate. Patient demographics and the levels operated on are listed in Table 1.

**Table 1:** Patient demographics and fusion levels

Patient ID	Gender	Age	BMI	Smoker	# of Levels	Levels
1	F	37	32	Yes	1	T5-6
2	M	57	28	No	1	T8-9
3	F	57	34	No	2	T8-10
4	F	36	37	No	2	T9-10, T11-12

### Inclusion criteria

Patients with herniated thoracic disk disease below T6, who had failed to respond to conservative therapy for four-six months and in whom discography had confirmed the origin of pain in the level of question were included. For all cases, a control level was studied during discography and was negative.

### Exclusion criteria

Patients with scoliosis, significant other vertebral anatomic abnormalities like hyperkyphosis or pathologies above T6 or where scapula was within approach were excluded.

### Operation room setup

**Figure 1:**
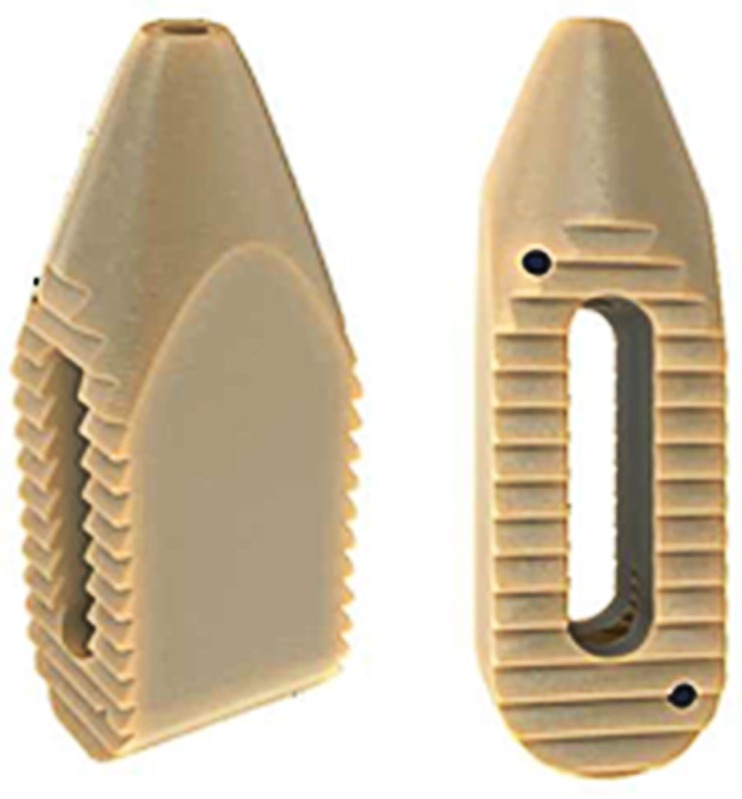
PEEK Zeus-O cage with bullet-nosed tip

The patient was placed in the prone position on the operating table tilted away from the surgeon by 3-5º until after the PEEK Zeus-O® cage (Amendia, Georgia, USA), (Figure 1) was inserted. After cage insertion, the patient was placed back into a true prone position. To enable quick readjustment 3M™ Ioban™ transparent plastic draping (3M Center, St. Paul, MN) was used to help the surgeon get a sense of the patient’s anatomy and positioning. The surgical setup is similar to the setup described in the OLLIF procedure [17].

For the setup of the bilateral fluoroscopy, it is important that the endplates of the target level line up well in the lateral view and the ribs are exactly leveled. In the anterior-posterior (AP) view, the disk needs to be visible and the spinous process should be centered between the pedicles. Electrophysiological monitoring was set up on the major muscle groups and the skull to monitor somatosensory evoked potentials, electromyogram, and transcranial motor evoked potentials throughout the surgery.

### Marking

**Figure 2:**
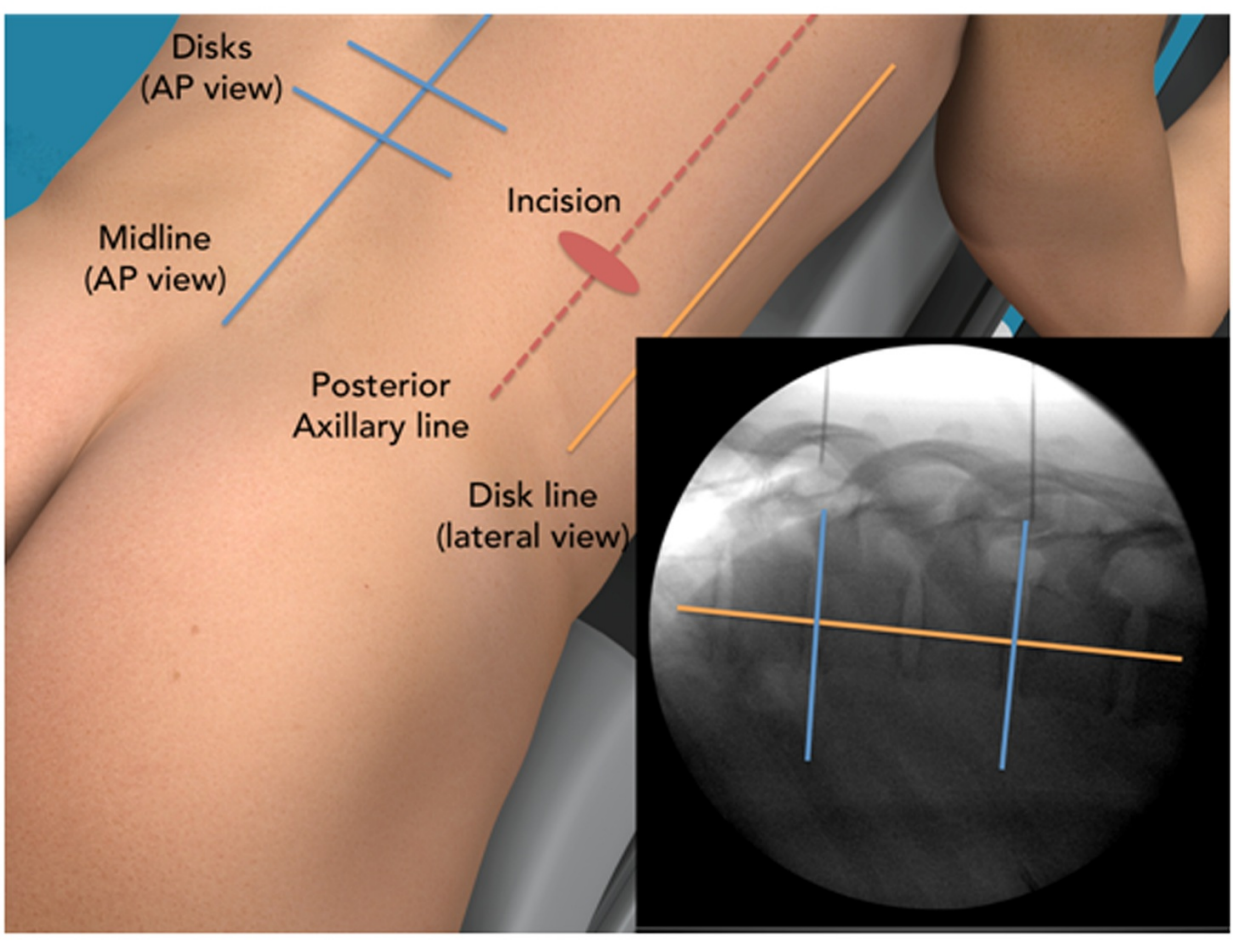
MIS-DTIF back region markings for incisions The incision point is chosen to give a 45º angle of approach to the spine.

The posterior axillary line was projected on the spinal level in question and lines were drawn on the back region (Figure 2). Using the AP view, the midline and each disk were marked. A vertical line showing the midpoint of each disk was marked using the lateral view. The incision was close to the posterior axillary line. Multiple levels can be approached through the same incision by shifting the skin to approach the disk at a slight angle. Although this mobility is reduced compared to the lumbar region due to the ribs, the skin can still be shifted to reach above and below the rib.

### Surgical approach

**Figure 3:**
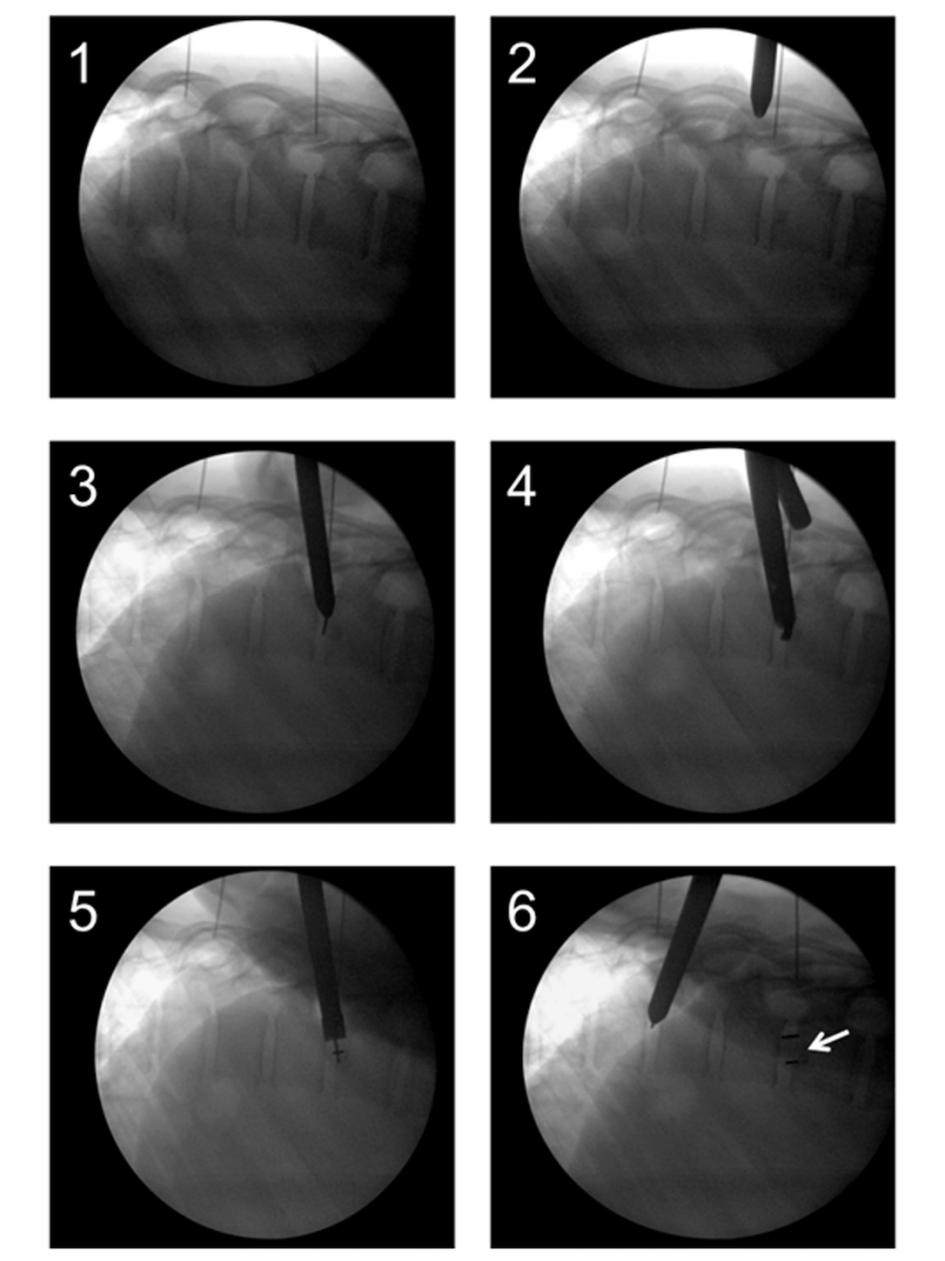
MIS-DTIF in the lateral fluoroscopic view 1) Localization, 2) Approach, 3) Disk entry, 4) Disk removal using drill, 5) Cage entry, 6) Cage entry completed (arrow).

The appropriate rib was identified and a 2-cm incision was placed just above the rib on the posterior axillary line. A blunt 8-mm cannulated probe was inserted into the pleural space and then “walked” along the rib while touching it at all time to the head of the rib within the pleural space. The probe was positioned directly on the side of the disk space of interest. The correct position was confirmed by biplanar fluoroscopic imaging as free hand placement is not reliable [18-19]. At this point, a K-wire with a sharp tip was introduced into the cannulated probe and entered into the disc space under biplanar fluoroscopic imaging. The probe was tapped with a mallet into the disc space and the K-wire was removed. Then, a working tube was placed over the blunt probe and entered into the disc space, thus creating a working channel from the skin into the disk space. Through the 10-mm working tube, a direct connection from the outside of the skin to the disk space was maintained throughout the surgery, sealed by skin and intercostal tissue. The approach in the lateral fluoroscopic view is shown in Figure 3, panels 1-2.

### Discectomy and cage placement

**Figure 4:**
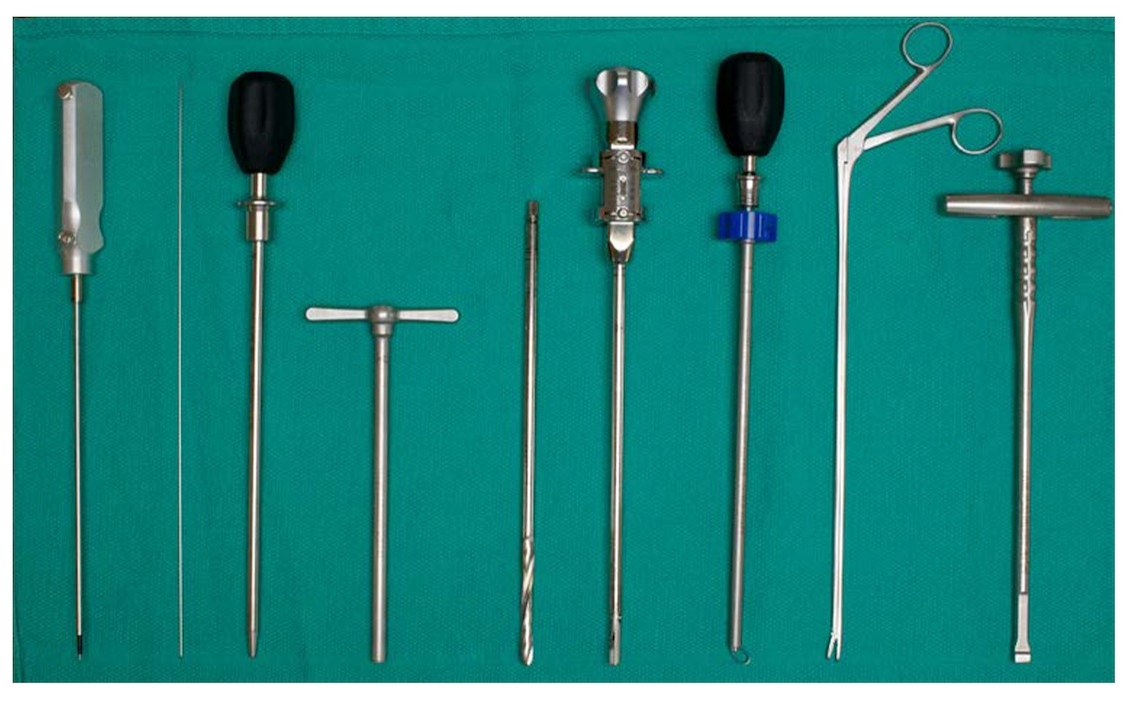
Instrumentation used for MIS-DTIF (Left to right): Probe, K wire, dilator, working tube, disk drill, rotating curette, ring curette, rongeur, cage insertion device.

Tools for discectomy were delivered through the working tube. Disk material was removed first with a drill and then with a rotating curette, ring curette, and rongeur. The tools used for MIS-DTIF can be seen in Figure 4. Next, the endplates were prepared with a gradual dilation of the rotating curette. Tactile feedback from the curette indicates when the endplates are reached and free through high frequency vibration. The disk space was packed with tricalcium phosphate (TCP, Berkeley Advanced Biomaterials Inc., Berkeley, CA) that had been soaked in autologous bone marrow aspirate drawn through a Jamshidi® needle (BD, New Jersey, USA) from one of the pedicles above the interested level [20]. The K-wire was once again placed in through the working tube and the tube was removed. Next, the cone-shaped PEEK Zeus-O cage was inserted over the K-wire and guided through the pleural space using biplanar fluoroscopic imaging. Once the cage was positioned on the top of the disc, it was entered into the disk space with mallet taps until one-third of the cage was past the midline. At this point, the insertion device was removed and the pleural space was sutured in two layers. The discectomy and cage placement in the lateral fluoroscopic view are in Figure 3, panels 3-6.

### Posterior pedicle screw fixation

After the cage placement was complete, we performed percutaneous posterior pedicle screw fixation. Savannah-T posterior instruments and high top screws manufactured by Amendia were used. Jamshidi needles had already been placed in one level at the beginning of the surgery to draw bone marrow aspirate. Now all remaining pedicles were tapped with Jamshidi needles that were stimulated at 30 mV, and accepted at 18 mV or above to assure there was no contact to neural structure. It is important to ensure all Jamshidi needles are positioned correctly because repositioning is easiest at this stage. K-wires were then placed through the Jamshidi needles and once the positioning of all K-wires was confirmed, the AP fluoroscopic arm was removed to ease screw placement. An osteotome was slid down the K-wire and the facets were bare boned aided by lateral fluoroscopy. A small amount of dry TCP was placed in the newly created space on the facets. Finally, screws were inserted and the rod was placed as described by Foley, similar to the lumbar technique [8].

### Outcome measures

Skin-to-skin surgery time, fluoroscopy time, and blood loss were measured by the principal investigator and recorded in the electronic medical records (EMR) database immediately after surgery by the clinic staff. Blood loss was measured by weighing sponges and subtracting the dry weight. All patients received chest x-rays (CXR) and CAT scans post-surgery to monitor potential complications. Initial follow-up was done 30 days post-surgery.

## Results

### Perioperative outcomes

In this group of four MIS-DTIF surgeries, two were single level fusions, and two were two-level fusions. For the single level surgery the mean surgery time was 43 minutes, blood loss was 90 ml, fluoroscopy time was 293 seconds, and the hospital stay for both patients was two days. For the two-level surgeries, the mean surgery time was 61 minutes, blood loss was 27 ml, fluoroscopy time was 321 seconds, and the hospital stay for both patients was three days. The complete perioperative outcomes are displayed in Table 2.

**Table 2:** Perioperative outcomes OP: Operating

Patient ID	Blood Loss (ml)	OP Time (mins)	Fluoro Time (s)	Hospital Stay (days)
1	125	41	247	2
2	55	45	339	2
3	15	76	226	3
4	38	45	416	3

### Complications

Routine thoracic CAT scans were performed for all patients. No complications were encountered during surgery. A hemothorax measuring 1 cm in diameter was found on the supine CAT scan of one patient post-surgery. It was observed with repeated CAT scans and did not require intervention. Apart from this, all patients were free from hemothorax, pleural effusion, and pneumothorax as confirmed by CXR and CAT scan. No patient required chest tube or drainage and no respiratory or cardiac problems were encountered.

### Patient reported pain

Before surgery, patients reported an average of 8.8 on a 10-point (1 through 10) sliding pain scale. A scoring of 1 represents no perceived pain and a scoring of 10 representing the maximum pain the patient could perceive. At the first follow-up (26-32 days post-surgery), pain was reduced significantly to a score of 3.5.

## Discussion

In the initial patient group, MIS-DTIF has been identified as a fast and clinically effective procedure. The operating room (OR) times for this procedure are lower than any previously reported OR times for fusions of the thoracic spine [17, 21]. No patient stayed in the hospital for more than three days and there were no complications apart from one clinically insignificant case of hemothorax. The early outcomes and the lack of complications show that MIS-DTIF is a promising procedure warranting further study.

Other thoracic approaches carry multiple risks such as lung contusion, direct mechanical trauma, and acute respiratory distress syndrome (ARDS) due to invasion of thoracic cavity and displacement or collapse of the lung to gain access to the spine [22-23]. In our MIS-DTIF approach, displacement or collapse of the lung is not necessary, hence the risk of these complications is reduced. In our experience, MIS-DTIF is technically less demanding than endoscopic approaches to the thoracic spine, though the learning curve for the surgeon is still steep.

MIS-DTIF enables the surgeon to freely pack the disc space with TCP or a biologic because the opening into the disc space is small and is essentially sealed by the cage [24-25]. We hypothesize that this leads to higher fusion rates [26] and lower rates of reoperation. We are currently collecting data to investigate this.

MI surgeries of the spine are often cheaper than open surgeries due to shorter hospitalization and reduced complication rates [2, 9-10]. In addition, MIS-DTIF is performed by a single surgeon while conventional approaches require a vascular and a thoracic surgeon [27-28]. Therefore, we hypothesize that MIS-DTIF reduces the overall cost to the health care system, compared to open surgeries. We plan to address the overall cost of MIS-DTIF in future studies.

This is a POC study for a novel approach to fusion of the thoracic spine. We are aware of the small sample size and are in the process of expanding the study cohort and collecting long term outcome data such as disability indices and fusion rates, but we believe that the significant benefit of this approach to patients warrants publication of the study and collaboration for a more thorough, possibly multi-center, study. We invite academic and non-academic centers to join us for a multicenter study. In addition, we are investigating the possibility of expanding this technique to higher disk levels of the thoracic spine and to more complex cases of spinal diseases.

## Conclusions

MIS-DTIF with pedicle screw fixation is a novel, minimally invasive approach to fusions of the thoracic spine as a treatment for disk herniation and degenerative disk disease. During this proof of concept study, we performed MIS-DTIF on six levels in four patients. In our experience, MIS-DTIF has been safe, clinically effective, and extremely fast, taking on average an hour or less for one- and two-level procedures. We have not encountered any clinically significant complications. We conclude that MIS-DTIF is a promising procedure that warrants further study and offers the potential for improved outcomes and cost savings. We are currently expanding this study to a larger cohort and collecting data regarding the long term outcomes and costs and invite other centers to join us for this investigation.
